# Investigating the relationship between language teachers’ occupational self-efficacy, satisfaction and meaning at work, and their subjective wellbeing

**DOI:** 10.3389/fpsyg.2023.1219130

**Published:** 2023-07-25

**Authors:** Samaneh Chamani, Farzaneh Safaeizadeh, Ismail Xodabande

**Affiliations:** ^1^Department of Foreign Languages, University of Guilan, Rasht, Gilan, Iran; ^2^Department of Foreign Languages, Islamic Azad University North Tehran Branch, Tehran, Alborz, Iran; ^3^Department of Foreign Languages, Kharazmi University, Tehran, Iran

**Keywords:** language teachers, occupational self-efficacy, satisfaction and meaning at work, subjective well-being, teacher psychology, language teacher wellbeing

## Abstract

Despite the growing interest in the well-being of educators, there is a significant gap in the literature regarding the specific factors that influence the well-being of language teachers. To address this gap, this study explored the relationship between Iranian language teachers’ occupational self-efficacy, satisfaction and meaning at work and their subjective well-being. The study involved 120 EFL teachers, and data were collected through self-report questionnaires. The results pointed to significant positive correlations between subjective well-being and occupational self-efficacy (r = 0.625, *p* < 0.001) as well as satisfaction and meaning at work (r = 0.493, *p* < 0.001). Regression analyses further indicated that occupational self-efficacy, satisfaction and meaning at work were significant predictors of subjective well-being. Notably, occupational self-efficacy emerged as a stronger predictor, outweighing the influence of satisfaction and meaning at work. Furthermore, the ANOVA results demonstrated that the regression models significantly contributed to the prediction of subjective well-being, indicating the relevance of these factors in understanding language teachers’ well-being. The coefficients analysis supported the significance of occupational self-efficacy (β = 0.625, *p* < 0.001) in predicting subjective well-being, while satisfaction and meaning at work also made a significant contribution (β = 0.258, *p* = 0.003). These findings suggest that enhancing teachers’ occupational self-efficacy, satisfaction and meaning at work could promote their subjective well-being. The study contributes to understanding the relationship between teachers’ job-related factors and their subjective well-being and could have implications for developing interventions to enhance their well-being.

## 1. Introduction

It is widely recognized that language teachers play a significant role in shaping their students’ linguistic competence. However, being a language teacher might be demanding, with a range of difficulties and challenges, including a high level of workload, low payments, and limited career and professional development opportunities, that may impact their well-being negatively (Mercer et al., 2016; MacIntyre et al., 2019, 2020; Gregersen et al., 2020; Mercer, 2020, 2021; Hofstadler et al., 2021; Jin et al., 2021; Shin et al., 2021; Sulis et al., 2021; Babic et al., 2022). In this regard, research has indicated that job satisfaction and meaning at work are crucial factors in determining employee well-being (Lavy and Bocker, 2018; Kun and Gadanecz, 2022), and occupational self-efficacy might also predict their job satisfaction and meaning at work (Peng and Mao, 2015). Therefore, investigating the relationship between these variables and subjective well-being among language teachers is of significance importance.

This study seeks to contribute to the existing literature by examining the relationship between these key variables and their impact on the subjective well-being of language teachers. The study is significant as understanding such relationships can provide valuable insights for promoting teacher wellbeing and enhancing the overall quality of language education. Moreover, findings of the study might have practical implications for language teacher education and development programs, as well as for school administrators who are responsible for creating work environments that foster job satisfaction, meaning at work, and self-efficacy beliefs among language teachers. Therefore, outcomes of this study may inform their decisions and practices to cultivate positive work environments that support teacher well-being. Furthermore, understanding the factors contributing to the subjective wellbeing of language teachers can also help inform policies and practices that promote teacher retention, job performance, and student achievement. By prioritizing teacher well-being, educational institutions can create a positive ripple effect throughout the educational system.

## 2. Literature review

### 2.1. Subjective wellbeing

Subjective well-being (SWB) refers to an individual’s subjective evaluation of their overall quality of life, which includes their emotional state, cognitive appraisal of their life circumstances, and overall life satisfaction (Diener and Ryan, 2009; Proctor, 2014; Das et al., 2020). SWB is a multidimensional construct that draws upon different theoretical approaches, such as the hedonic and eudaimonic perspectives, to capture the subjective experiences and evaluations of individuals’ well-being. The hedonic perspective of subjective well-being emphasizes the role of positive and negative affect in shaping individuals’ overall well-being (Lyubomirsky et al., 2005; Magyar and Keyes, 2019). Positive affect refers to the experience of positive emotions, including happiness, joy, and contentment, while negative affect encompasses negative emotions such as sadness, anxiety, and anger (Proctor, 2014). The hedonic approach recognizes the importance of experiencing positive emotions and minimizing negative emotions as key components of subjective well-being. In addition to affective experiences, subjective well-being also includes the cognitive evaluation of one’s life circumstances, known as life satisfaction (Shin and Johnson, 1978). Life satisfaction involves an individual’s cognitive appraisal of their overall fulfillment, purpose, and satisfaction with various domains of life (e.g., work, relationships, health; Proctor, 2014). This cognitive dimension of subjective well-being allows individuals to reflect on their life circumstances and make judgments about their overall satisfaction with their lives. Subjective well-being is a critical aspect of human functioning and has been linked to a range of positive outcomes, such as better physical health, greater resilience, improved social relationships, and better work performance (Proctor, 2014; Das et al., 2020).

### 2.2. Occupational self-efficacy and well-being

Occupational self-efficacy refers to an individual’s belief in their ability to successfully perform tasks and handle challenges within their specific occupational domain (Felfe and Schyns, 2006). It is the degree to which an individual feels confident in their abilities to accomplish their job responsibilities and achieve their career goals (Peng et al., 2021). Occupational self-efficacy can influence an individual’s level of motivation, job satisfaction, and overall performance in the workplace (Wallin et al., 2022). People with high occupational self-efficacy tend to set challenging goals, persist in the face of obstacles, and view setbacks as temporary and surmountable (Kun and Gadanecz, 2022). In contrast, individuals with low occupational self-efficacy may be more likely to avoid challenging tasks or give up quickly when faced with difficulties (Barouch Gilbert et al., 2014; Peng and Mao, 2015). In language teaching, occupational self-efficacy is important and relevant for several reasons. First, language teachers with high occupational self-efficacy are more likely to have better control over their classroom, manage disruptive behavior, and create a positive learning environment for their students (Kurt et al., 2014). Second, teachers who believe in their ability to teach a particular language are more likely to engage in effective instructional practices, such as differentiated instruction, scaffolding, and timely and constructive feedback (Polatcan et al., 2021). Third, competent and effective teachers are more likely to experience job satisfaction and commitment, leading to better job performance and retention (Türkoğlu et al., 2017). Moreover, occupational self-efficacy can also influence a language teacher’s willingness to engage in professional development activities (Malmir and Mohammadi, 2018).

A number of studies have investigated the relationship between occupational self-efficacy and subjective well-being. These studies have shown that higher levels of occupational self-efficacy are positively associated with greater subjective well-being among educators and other professionals. For example, a study by Bandura and Locke (2003) found that teachers with high levels of occupational self-efficacy reported higher levels of job and overall life satisfaction. Similarly, a study by Scholz et al. (2002) found that occupational self-efficacy was positively related to work-related stress, with individuals with higher levels of self-efficacy experiencing lower stress levels and greater overall well-being. Other studies have explored the potential negative impact of low occupational self-efficacy on subjective well-being. For example, a study by Betoret (2006) found that low self-efficacy among teachers was associated with increased levels of burnout and reduced job satisfaction.

Some studies have also examined the role of self-efficacy beliefs in enhancing or undermining well-being among educators specifically. A study by Wubbels et al. (1991) found that teachers with higher levels of self-efficacy were more effective at managing student behavior and had greater job satisfaction than those with lower levels of self-efficacy. Similarly, a study by Tschannen-Moran and Hoy (2007) found that self-efficacy beliefs were a significant predictor of teacher job satisfaction and that teachers with high levels of self-efficacy were more likely to persist in their careers and experience greater overall well-being. However, not all studies have found a significant relationship between occupational self-efficacy and subjective well-being. For example, a study by Bakker et al. (2004) found that self-efficacy beliefs did not significantly predict work engagement or burnout among healthcare professionals. Despite some mixed findings, most research supports the notion that occupational self-efficacy is an essential predictor of subjective well-being among educators and other professionals. Self-efficacy beliefs can impact an individual’s motivation, resilience, and ability to cope with job-related stressors, affecting their overall well-being and job satisfaction. However, it is important to note that self-efficacy beliefs are not static and can be influenced by various factors, including organizational culture, job demands, and social support.

### 2.3. Job satisfaction and well-being

Satisfaction at work refers to an individual’s overall positive feeling or level of contentment with their job or work environment (Thompson and Phua, 2012). It is a subjective evaluation of one’s job-related experiences and can be influenced by factors such as job autonomy, work relationships, job security, compensation, and work-life balance (Mohammed et al., 2022; Eriksson et al., 2023). For several reasons, job satisfaction is extremely important for language teachers (Sadeghi et al., 2021). When teachers are satisfied with their jobs, they are more motivated to perform well and to improve their teaching skills (Strydom et al., 2011). This, in turn, leads to better outcomes for their students (Demirtas, 2010). Moreover, job satisfaction is also critical for retaining teachers in the profession. When teachers are unhappy with their jobs, they are more likely to leave the profession, resulting in a shortage of experienced teachers and a negative impact on student learning (Appiah-Agyekum et al., 2013). Furthermore, job satisfaction has been linked to better mental health and well-being. When teachers are satisfied with their jobs, they are less likely to experience burnout or stress-related illnesses (Benevene et al., 2018). Additionally, satisfied teachers are more likely to be engaged in their work and to put forth their best effort. This can lead to improved job performance and better student outcomes (Day and Qing, 2009; Acton and Glasgow, 2015; MacIntyre et al., 2019).

Job satisfaction has long been recognized as an important factor in employee well-being, with numerous studies exploring the relationship between job satisfaction and various dimensions of subjective well-being. Studies have consistently shown a positive relationship between job satisfaction and psychological well-being. For example, a meta-analysis of 223 studies found that job satisfaction was strongly associated with higher levels of subjective well-being, with the relationship being particularly strong for measures of life satisfaction and positive affect (Judge et al., 2013). One potential explanation for this relationship is that job satisfaction may contribute to a sense of mastery and control over one’s environment, which can lead to a greater sense of well-being (Peng and Mao, 2015; Steger et al., 2019). Job satisfaction may also lead to greater engagement and commitment to one’s work, promoting feelings of accomplishment and self-esteem (May et al., 2004).

Studies have found that job satisfaction positively relates to social well-being, particularly work-related relationships. For example, a study of healthcare workers found that job satisfaction was associated with higher levels of perceived social support from colleagues, which in turn was related to lower levels of burnout and greater job engagement (Cañadas-De la Fuente et al., 2015). Job satisfaction may also promote social well-being by enhancing one’s ability to form and maintain positive relationships outside work. For example, a study of Australian employees found that job satisfaction was positively associated with higher levels of overall social support and greater satisfaction with family and leisure time (Cummins et al., 2002).

Overall, the literature suggests that job satisfaction is an important predictor of subjective well-being, with positive relationships observed across psychological, social, and physical dimensions. While these relationships’ mechanisms are complex and multifaceted, they may be related to factors such as control over one’s environment, social support from colleagues, and reduced stress levels. It is important for employers to recognize the importance of job satisfaction for employee well-being and to promote job satisfaction through factors such as supportive leadership, fair compensation, and opportunities for career growth and development.

### 2.4. Meaning at work and well-being

Meaningful work has been shown to have a positive impact on subjective well-being. Work-related meaning has been defined as “the degree to which individuals experience their work as significant, purposeful, and valuable” (Rosso et al., 2010, p. 90). Moreover, meaning at work refers to an individual’s sense of purpose and fulfillment in their job or work environment (Vuori et al., 2012). It is a more profound and intrinsic aspect of work, reflecting an individual’s perception that their work has significance and contributes to a larger goal or purpose beyond themselves (Whittington et al., 2017). While job satisfaction and meaning at work are related, they are distinct concepts. It is possible to be highly satisfied with one’s job but still feel a lack of meaning or purpose, and vice versa (Whittington et al., 2017). Research suggests that experiencing a sense of meaning at work is associated with a range of positive outcomes, such as higher job satisfaction, greater engagement, better mental health, and lower rates of burnout (Barouch Gilbert et al., 2014; Kun and Gadanecz, 2022).

Furthermore, research has consistently shown that individuals who experience a sense of meaning at work report higher subjective well-being levels (Martela and Steger, 2016). Demerouti et al. (2001) examined the relationship between work-related meaning and burnout among healthcare professionals. The results showed that individuals who reported a higher sense of meaning in their work experienced less burnout, indicating that work-related meaning may serve as a protective factor against burnout. Another study by Martela and Steger (2016) explored the relationship between work-related meaning and subjective wellbeing among educators. The results showed that teachers who reported a higher sense of meaning in their work reported greater life satisfaction, psychological wellbeing, and lower levels of emotional exhaustion. Additionally, research reveals that teachers’ sense of meaning contributes to students’ perception of being cared for and wellbeing too (Lavy and Naama-Ghanayim, 2020).

However, it is important to note that the relationship between work-related meaning and subjective well-being may be influenced by factors such as job demands and resources, organizational culture, and personal values and goals (Martela and Steger, 2016). For example, a study by Harzer and Ruch (2014) examined the relationship between work-related meaning and teacher job satisfaction and found that personal values moderated the relationship. Specifically, individuals who placed a high value on self-transcendence (i.e., concern for the welfare of others) reported a stronger relationship between work-related meaning and job satisfaction than individuals who placed a lower value on self-transcendence. Another study by Steger et al. (2012) explored the role of work-related meaning in promoting well-being among employees in a non-profit organization. The results showed that work-related meaning was positively associated with job satisfaction and mental health. However, this relationship was stronger for employees who reported higher job resources (e.g., autonomy, social support) and lower job demands (e.g., workload, time pressure).

These studies suggest that the relationship between work-related meaning and subjective well-being may be complex and may depend on various individual and contextual factors. Research also suggests that work-related meaning is important in promoting subjective well-being among educators and other professionals. Individuals who experience a sense of meaning in their work report higher levels of life satisfaction, job satisfaction, and mental health, as well as lower levels of burnout and emotional exhaustion. However, job demands, resources, organizational culture, and personal values and goals may influence the relationship between work-related meaning and subjective well-being. Overall, findings highlight the importance of considering work-related meaning as a potential factor in promoting subjective well-being among professionals.

### 2.5. The present study

The literature review indicated that occupational self-efficacy, satisfaction and meaning at work, and subjective well-being are all interrelated concepts that can influence each other in various ways. Accordingly, teachers with high occupational self-efficacy tend to experience higher job satisfaction and a greater sense of meaning at work. Conversely, low levels of job satisfaction and meaning at work can lead to negative emotions and lower levels of life satisfaction. Although research on teachers’ occupational self-efficacy, satisfaction and meaning at work, and subjective well-being has gained attention in education, gaps still need to be addressed. Firstly, most studies have focused on the relationship between these variables and job performance rather than subjective well-being, which is a relatively new and underexplored concept in education (Mercer, 2021; Weiland, 2021). Secondly, most studies have been conducted in Western countries and have primarily focused on teachers working in developed countries. Accordingly, there is a need to investigate this relationship in different contexts, such as non-Western and developing countries, where the cultural and institutional contexts may differ. Lastly, language teachers’ well-being remained neglected in this research line compared to expanding body of knowledge in general education (MacIntyre et al., 2020; Mercer, 2020; Hofstadler et al., 2021; Jin et al., 2021; Sulis et al., 2021). To address part of this gap in the literature, the current study aimed to answer the following research questions:

1. How are Iranian language teachers’ occupational self-efficacy, satisfaction and meaning at work, and subjective well-being related?
2. How do occupational self-efficacy, satisfaction, and meaning at work predict language teachers’ SWB?

## 3. Methods

### 3.1. Participants and the study context

The study’s sample comprised 120 teachers, consisting of 40 males and 80 females, aged 19 to 54 years (M = 31). The participants had varying levels of teaching experience ranging from 3 to 20 years. Regarding educational qualifications, 45 participants held a BA in TEFL, 63 held an MA, and 12 held a Ph.D. The participants provided informed consent before their inclusion in the study. Overall, the study involved a diverse and heterogeneous sample of language teachers, comprising individuals with differing levels of education and experience and incorporating both genders. Concerning the study context, English is taught as a compulsory subject from the first year of primary school, and students study it until the end of high school. ELT in Iran faces several challenges, such as inadequate materials and resources, insufficient teacher training, and a lack of exposure to English outside the classroom (Borjian, 2013).

Additionally, cultural differences and students’ low motivation toward English learning can also challenge the ELT process (Khajavi and Abbasian, 2011; Sadeghi and Sepahi, 2018; Tajeddin and Chamani, 2020). Despite these challenges, the Iranian government has tried to improve ELT in the country. These include the development of textbooks and curriculum guidelines, teacher training programs, and establishing language schools and institutes (Tajeddin and Chamani, 2020). In recent years, Iran’s ELT has witnessed significant progress, introducing new teaching methods, innovative materials, and more focus on communicative language teaching (Babaii, 2022). Additionally, the demand for English proficiency has increased due to globalization, which has led to a surge in the number of language schools and institutes across the country (Sadeghi and Richards, 2016). ELT in Iran continues to evolve and develop, with efforts underway to overcome the challenges faced by the system and provide quality education to students (Rassouli and Osam, 2019; Babaii, 2022).

### 3.2. Instruments

#### 3.2.1. Personal efficacy beliefs scale

In order to measure the teachers’ occupational self-efficacy, the study used Personal Efficacy Beliefs Scale (PEBS), a self-report scale that assesses an individual’s belief in their personal work skills and ability to perform their job. Developed by Riggs et al. (1994) as part of their study on the development and validation of self-efficacy and outcome expectancy scales for job-related applications, the scale consists of 10 items, each rated on a 6-point Likert-type scale ranging from 1 (strongly disagree) to 6 (strongly agree). The items on the scale assess various aspects of an individual’s self-efficacy beliefs, including their confidence in their ability to do their job (item 1), their perceived level of skill (item 5), and their belief in their prospects in their job (item 8). The scale also includes items that tap into potential barriers to self-efficacy, such as feeling threatened by others watching them work (item 10) and doubts about their ability to perform their job (item 4). Overall, the PEBS provides a reliable and valid measure of an individual’s self-efficacy beliefs related to their job performance (O’Brien et al., 2019). The scale’s internal consistency, or reliability, was evaluated using Cronbach’s alpha coefficient. In the present study, the Cronbach’s alpha coefficient for the PEBS was found to be 0.76, indicating an acceptable level of internal consistency.

#### 3.2.2. The work and meaning inventory

The study used work and meaning inventory (WAMI) to measure satisfaction and meaning in work among language teachers. This self-reported survey measures an individual’s perception of the meaning and purpose of their work or career (Steger et al., 2019). The survey consists of 10 items, and respondents are asked to rate how well each statement applies to them and their work using a 5-point Likert scale. The scale ranges from “absolutely untrue” to “absolutely true,” with a neutral response option of “neither true nor untrue/cannot say.” The survey assesses three subscales of meaningful work: Positive Meaning, Meaning-Making Through Work, and Greater Good Motivations. The Positive Meaning subscale measures the extent to which an individual finds their work fulfilling and meaningful. The Meaning-Making Through Work subscale assesses how an individual’s work contributes to their personal growth and development. The Greater Good Motivations subscale evaluates the degree to which an individual’s work serves a larger purpose beyond personal fulfillment. Accordingly, the WAMI survey provides a way to understand an individual’s perception of the meaning and purpose of their work or career (Steger et al., 2019). The internal consistency of the WAMI was evaluated using Cronbach’s alpha coefficient. In this study, the Cronbach’s alpha coefficient for the WAMI was found to be 0.72, indicating an acceptable level of internal consistency.

#### 3.2.3. Subjective well-being scale

The study used Subjective Well-being Measure (Magyar and Keyes, 2019) that is designed to assess the frequency with which a person experiences various positive feelings and attitudes. The scale comprises 12 items that ask respondents to report how often they have felt happy, interested in life, satisfied, etc. Respondents are asked to rate the frequency of these experiences using a 5-point Likert scale ranging from never to almost every day to every day. Moreover, the items on this scale are designed to assess a wide range of positive feelings, attitudes, and experiences that are thought to contribute to overall well-being. For example, the items that assess feelings of happiness, interest in life, and satisfaction with life are designed to capture a person’s overall level of positive affect. Other items, such as the items that assess feelings of belonging to a community, are designed to assess a person’s sense of social connectedness and trust in others. Finally, the items that assess feelings of confidence in managing daily responsibilities, having warm relationships with others, and expressing one’s own ideas and opinions are designed to assess a person’s sense of agency and autonomy. By asking respondents to rate the frequency of these experiences over the past month, the scale provides a useful snapshot of a person’s overall sense of well-being and can be used to track changes in well-being over time (Magyar and Keyes, 2019). The scale’s internal consistency was evaluated using Cronbach’s alpha coefficient, which in this study yielded a value of 0.83, indicating a high level of internal consistency and reliability.

### 3.3. Procedures and data analysis

The research procedures for investigating the relationship between language teachers’ occupational self-efficacy, satisfaction and meaning at work, and their subjective well-being involved using an online survey. It is worth noting that participants in the study were invited through social media and email, and the response rate was 68%. A response rate of 68% is relatively high for an online survey, which suggests that the participants were likely motivated to complete the survey and were representative of the larger population of Iranian language teachers. However, it is important to acknowledge that there may be some selection bias in the sample, as individuals who choose to participate in research studies may differ from those who do not. The data collected from the survey was analyzed using statistical techniques of correlation and hierarchical regression analysis to determine the strength and direction of the relationship between the variables under investigation. Hierarchical regression analysis is a statistical technique used to examine the incremental contribution of predictor variables in predicting an outcome variable (Pallant, 2016). It involves a stepwise approach where predictor variables are entered into the regression equation in a predetermined order. The method allows researchers to assess the unique variance explained by each variable, controlling for the effects of previously entered variables. By systematically adding variables, hierarchical regression analysis helps understand the relative importance of predictors and their impact on the outcome variable, providing insights into the hierarchical structure of the relationships.

## 4. Results

According to the descriptive statistics, the participants reported moderate levels of occupational self-efficacy (M = 39.37, SD = 7.206), satisfaction and meaning at work (M = 36.73, SD = 5.072), and relatively high levels of subjective well-being (M = 77.82, SD = 9.088). The skewness values for all variables were negative, indicating a slightly skewed distribution. The kurtosis values for occupational self-efficacy and satisfaction and meaning at work were negative (−0.925 and − 0.943, respectively), indicating a platykurtic distribution, which is flatter and has fewer extreme values than a normal distribution. The kurtosis value for subjective well-being was close to zero (0.472), indicating a mesokurtic distribution similar to a normal distribution (Table 1).

**Table 1:** Descriptive statistics.

	*N*	Mean	Std. deviation	Skewness		Kurtosis	
Occupational self-efficacy	120	39.37	7.206	−0.176	0.238	−0.925	0.472
Satisfaction and meaning at work	120	36.73	5.072	−0.038	0.238	−0.943	0.472
Subjective well-being	120	77.82	9.088	−0.264	0.238	−0.540	0.472

Table 2 presents the Pearson correlations among occupational self-efficacy, satisfaction and meaning at work, and subjective well-being based on a sample of 120 Iranian language teachers. The results indicated that there is a significant positive correlation between occupational self-efficacy and satisfaction and meaning at work (r = 0.465, *p* < 0.01) as well as between occupational self-efficacy and subjective well-being (r = 0.625, *p* < 0.01). Similarly, there is a significant positive correlation between satisfaction and meaning at work and subjective well-being (r = 0.493, *p* < 0.01). Overall, these findings suggest that Iranian language teachers’ occupational self-efficacy, satisfaction and meaning at work, and subjective well-being are interrelated.

**Table 2:** Correlations among subjective well-being, occupational self-efficacy, and satisfaction and meaning at work.

	Subjective well-being	Occupational self-efficacy	Satisfaction and meaning at work
Subjective well-being	1.000		
Occupational self-efficacy	0.625^*^	1.000	
Satisfaction and meaning at work	0.493^*^	0.465^*^	1.000

*N* = 120. Pearson’s correlation coefficients are reported in the table. ^*^p < 0.05 (two-tailed).

The results of the hierarchical regression analysis revealed important insights into the relationship between the predictors (occupational self-efficacy and satisfaction and meaning at work) and the dependent variable (subjective well-being). Two models were examined to determine the impact of these predictors on subjective well-being (Table 3).

**Table 3:** Model summary^c^.

Model	R	R square	Adjusted R square	Std. error of the estimate	Change statistics				
					R square change	F change	df1	df2	Sig. F change
1	0.625^a^	0.391	0.385	7.126	0.391	64.874	1	119	0.000
2	0.666^b^	0.443	0.432	6.848	0.052	9.364	1	118	0.003

^a^Predictors: (constant), occupational self-efficacy. ^b^Predictors: (constant), occupational self-efficacy, satisfaction and meaning at work. ^c^Dependent variable: subjective well-being.

In Model 1, which included only occupational self-efficacy as a predictor, the model accounted for a significant proportion of the variance in subjective well-being (R^2^ = 0.391, adjusted R^2^ = 0.385, *p* < 0.001). The correlation coefficient (R) indicated a moderate positive relationship between occupational self-efficacy and subjective well-being (R = 0.625). Model 2 expanded on Model 1 by including both occupational self-efficacy and satisfaction and meaning at work as predictors. This extended model provided a better fit, explaining a larger proportion of the variance in subjective well-being (R^2^ = 0.443, adjusted R^2^ = 0.432, *p* = 0.003). The correlation coefficient for Model 2 (R = 0.666) indicated a stronger positive relationship between the predictors and subjective well-being compared to Model 1. The F statistic for both models was statistically significant, indicating that the models as a whole significantly contributed to the prediction of subjective well-being [Model 1: *F*(1, 119) = 64.874, *p* < 0.001; Model 2: *F*(2, 118) = 9.364, *p* = 0.003]. The change in R^2^ between the two models was also significant [ΔR^2^ = 0.052, Δ*F*(1, 118) = 9.364, *p* = 0.003], suggesting that the inclusion of satisfaction and meaning at work as an additional predictor improved the model’s ability to explain variance in subjective well-being.

Table 4 shows the results of an analysis of variance (ANOVA), which is used to assess the significance of the predictors in the linear regression model. The results indicated that the regression models were significant in predicting subjective well-being. Model 1, including occupational self-efficacy as the predictor, showed a significant relationship with subjective well-being [*F*(1, 119) = 64.874, *p* < 0.001]. This model accounted for a substantial amount of the variance in subjective well-being. Model 2, which added satisfaction and meaning at work as a predictor, also demonstrated a significant relationship with subjective well-being [*F*(2, 118) = 39.805, *p* < 0.001]. The inclusion of these additional predictors explained a significant amount of additional variance.

**Table 4:** ANOVA^a^.

Model		Sum of squares	df	Mean square	F	Sig.
1	Regression	3294.463	1	3294.463	64.874	0.000^b^
	Residual	5129.033	119	50.783		
	Total	8423.495	120			
2	Regression	3733.615	2	1866.808	39.805	0.000^c^
	Residual	4689.880	119	46.899		
	Total	8423.495	120			

^a^Dependent variable: subjective well-being. ^b^Predictors: (constant), occupational self-efficacy. ^c^Predictors: (constant), occupational self-efficacy, satisfaction and meaning at work.

Table 5 reports the results for models coefficients. The regression coefficients provided important insights into the relationship between the predictors. In Model 1, occupational self-efficacy significantly correlated with subjective well-being (β = 0.625, *p* < 0.001). This indicates that higher levels of occupational self-efficacy were associated with higher levels of subjective well-being. In Model 2, the coefficient for occupational self-efficacy remained significant (β = 0.505, *p* < 0.001), indicating that it continued to be a strong predictor of subjective well-being. Additionally, satisfaction and meaning at work emerged as a significant predictor (β = 0.258, *p* = 0.003). This suggests that both occupational self-efficacy and satisfaction and meaning at work play a role in influencing subjective well-being.

**Table 5:** Coefficients^a^.

Model		Unstandardized coefficients		Standardized coefficients	t	Sig.	Correlations			Collinearity statistics	
		B	Std. error	Beta			Zero-order	Partial	Part	Tolerance	VIF
1	(Constant)	46.765	3.919		11.934	0.000					
	Occupational self-efficacy	0.789	0.098	0.625	8.054	0.000	0.625	0.625	0.625	1.000	1.000
2	(Constant)	35.752	5.209		6.863	0.000					
	Occupational self-efficacy	0.637	0.106	0.505	5.994	0.000	0.625	0.514	0.447	0.783	1.277
	Satisfaction and meaning at work	0.462	0.151	0.258	3.060	0.003	0.493	0.293	0.228	0.783	1.277

^a^Dependent variable: subjective well-being.

The correlations between the predictors and the dependent variable were consistent with the standardized coefficients. Occupational self-efficacy showed a strong positive correlation with subjective well-being (r = 0.625, *p* < 0.001), while satisfaction and meaning at work had a moderate positive correlation (r = 0.493, *p* < 0.001). There were no issues of multicollinearity, as indicated by the tolerances and variance inflation factors (VIF) within acceptable ranges (all VIF values < 1.277). Overall, these findings suggest that both occupational self-efficacy and satisfaction and meaning at work are important factors in understanding subjective well-being among teachers. Higher levels of occupational self-efficacy and satisfaction and meaning at work were associated with higher levels of subjective well-being.

**Figure 1:**
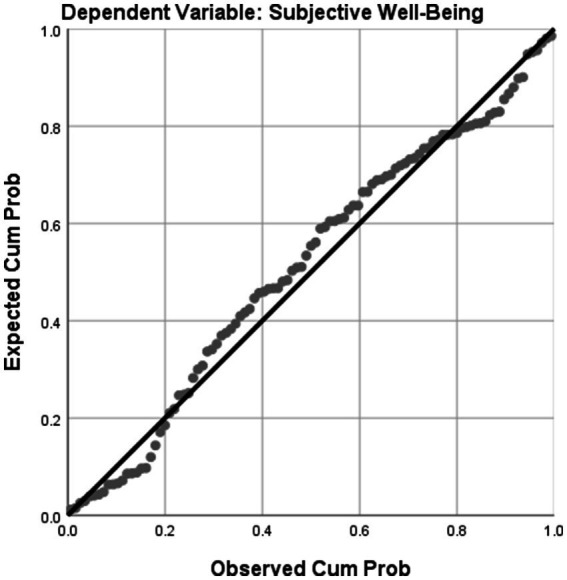
Normal P-P plot of regression standardized residual.

Overall, regression analysis results revealed that both occupational self-efficacy and satisfaction and meaning at work were significant predictors of subjective well-being (Figure 1). In the first regression model, which included only occupational self-efficacy as a predictor, the adjusted R-squared was 0.385. This means that approximately 38.5% of the variance in subjective well-being could be explained by occupational self-efficacy alone. In the second regression model, which included both occupational self-efficacy and satisfaction and meaning at work as predictors, the adjusted R-squared increased to 0.432. This indicates that the addition of satisfaction and meaning at work as a predictor increased the proportion of explained variance in subjective well-being by approximately 4.7%. Therefore, collectively, occupational self-efficacy and satisfaction and meaning at work accounted for approximately 43.2% of the variance in subjective well-being.

## 5. Discussion

Motivated by the existing gap in the literature on teachers’ subjective well-being, this study looked into the relationship between occupational self-efficacy, job satisfaction and meaning at work with teachers’ subjective well-being. It also attempted to explore which of the above-mentioned factors is a stronger predictor of teachers’ subjective well-being. Collecting data from 120 teachers working in Iranian EFL context, the findings revealed that teachers’ subjective well-being is directly related to the two factors, i.e., occupational self-efficacy and job satisfaction and meaning at work. In other words, teachers with higher levels of occupational self-efficacy and job satisfaction and meaning at work reported positive subjective well-being. In response to the first research question, the findings supported the ones illustrated in the existing research. More specifically, the positive correlation between occupational self-efficacy and subjective well-being aligns with Bandura’s social cognitive theory, which suggests that individuals who possess a strong belief in their capabilities are more likely to experience positive well-being outcomes. This finding is consistent with prior studies conducted in different educational contexts (e.g., Peng et al., 2021) and supports the notion that self-efficacy plays a crucial role in shaping teachers’ well-being. Moreover, the positive association between job satisfaction, meaning at work, and subjective well-being resonates with the broader literature on work and well-being. It aligns with theories such as the Job Demands-Resources model (Demerouti et al., 2001), which emphasizes the importance of satisfying work conditions and finding meaning in one’s job for enhanced well-being. Our findings add to the growing body of evidence suggesting that job satisfaction and meaning at work are important factors in promoting subjective well-being among teachers (Tschannen-Moran and Hoy, 2007; Türkoğlu et al., 2017; Malmir and Mohammadi, 2018). The present study also highlights the multidimensional nature of teachers’ subjective well-being, as it is influenced by multiple factors, including occupational self-efficacy, job satisfaction, and meaning at work. This finding is consistent with previous research emphasizing the complex and interactive nature of well-being in educational settings (Hakanen et al., 2006; Martinez-Alba and Herrera, 2021).

Regarding the second research question, the regression analysis findings shed light on the predictive power of occupational self-efficacy, satisfaction, and meaning at work on language teachers’ subjective well-being. Consistent with previous research (Tschannen-Moran and Hoy, 2007; Türkoğlu et al., 2017; Malmir and Mohammadi, 2018), our results indicate that these factors significantly contribute to teachers’ overall well-being. Notably, the analysis revealed that occupational self-efficacy emerged as a stronger predictor of subjective well-being compared to satisfaction and meaning at work. This aligns with earlier studies emphasizing the importance of teachers’ confidence in their abilities to perform their job duties (Tschannen-Moran and Hoy, 2007; Malmir and Mohammadi, 2018). When teachers feel competent and capable in their roles, they are more likely to experience higher levels of subjective well-being. The findings suggest that while job satisfaction and finding meaning in work are important, they may not have as pronounced an impact on teachers’ well-being as their perceived self-efficacy. This highlights the significance of teachers’ self-beliefs in their ability to meet the challenges of their profession and suggests that interventions aimed at enhancing self-efficacy can positively influence well-being outcomes (Türkoğlu et al., 2017). Theoretical perspectives on motivation further support these findings. For example, according to self-determination theory (Ryan and Deci, 2017), teachers who feel efficacious are more likely to experience intrinsic motivation, engagement, and a sense of accomplishment, all of which contribute to subjective well-being (Nazari and Xodabande, 2022). Consequently, fostering teachers’ self-efficacy beliefs becomes crucial in promoting their well-being and job satisfaction.

Research has consistently demonstrated that teachers’ happiness and well-being can significantly impact their students’ academic performance and well-being. When teachers experience negative subjective well-being can set the ground for a negative learning environment and lead to decreased student engagement, academic performance, and overall well-being. On the other hand, when teachers are happy and fulfilled in their work, they are more likely to create a positive classroom environment and establish supportive relationships with their students. Moreover, research has shown that teachers’ well-being is important for the students’ academic outcomes and plays a critical role in shaping the students’ social–emotional development (Martinez-Alba and Herrera, 2021). Happy and satisfied teachers with their job tend to model positive behaviors, attitudes, and emotions, which can impact the students’ emotional regulation, resilience, and social skills. Therefore, schools and educational institutions need to prioritize the well-being of their teachers and provide them with the necessary resources and support to maintain their happiness and fulfillment. By doing so, schools can create a positive and supportive learning environment that fosters academic and social–emotional development, leading to better outcomes for teachers and students.

The study’s findings have important practical implications for enhancing the subjective well-being of language teachers. The positive correlations observed between occupational self-efficacy, satisfaction and meaning at work, and subjective well-being highlight the potential avenues for intervention to promote teachers’ well-being. Firstly, efforts to increase teachers’ sense of self-efficacy in their profession can significantly impact their well-being. Providing teachers with professional development opportunities that enhance their skills and knowledge can boost their confidence and competence in their teaching practices. Moreover, offering support and mentorship programs that help teachers overcome challenges and develop strategies for success can further enhance their self-efficacy beliefs. By investing in these initiatives, educational institutions can empower teachers to feel more capable and effective in their roles, ultimately contributing to their subjective well-being. Secondly, fostering a work environment that promotes job satisfaction is crucial for enhancing teachers’ well-being. This can be achieved through various means, such as recognizing and appreciating teachers’ efforts and accomplishments, providing professional growth and advancement opportunities, and fostering positive relationships among colleagues. Creating a supportive and positive school culture that values teachers’ contributions and well-being can promote job satisfaction and, consequently, subjective well-being.

Additionally, the study emphasizes the importance of meaning at work in relation to teachers’ well-being. Offering opportunities for teachers to engage in meaningful work, such as involving them in curriculum development or decision-making processes, can enhance their sense of purpose and fulfillment. Providing a platform for teachers to contribute their unique perspectives and expertise can improve job satisfaction and overall well-being. Moreover, the finding that occupational self-efficacy had a stronger effect on subjective well-being compared to satisfaction and meaning at work highlights the importance of prioritizing efforts to enhance teachers’ self-efficacy beliefs. Providing targeted interventions and support systems that specifically focus on building teachers’ self-confidence can significantly impact their overall well-being. This can involve coaching and mentoring programs, reflective practices, and opportunities for peer collaboration, all of which can contribute to fostering self-efficacy. Finally, the implications of this study extend beyond the well-being of language teachers themselves. Creating a positive and supportive work environment for teachers can have far-reaching effects on students’ educational outcomes. Research has consistently shown that teacher well-being is linked to student engagement, achievement, and overall school climate (Sulis et al., 2023). By prioritizing teachers’ well-being and addressing factors such as occupational self-efficacy, satisfaction, and meaning at work, educational institutions can create an environment that supports teachers and positively impacts student learning and development.

## 6. Conclusion

The present study showed that the subjective well-being of Iranian EFL teachers is bound to the three main predicted variables: occupational self-efficacy, job satisfaction and meaning. The results underscore the importance of teachers’ subjective well-being in enhancing the quality of teaching. Teachers’ subjective well-being by itself is also tied to the positive condition available in the social context. Accordingly, if teachers are equipped with belief-boosting motivators and if they are situated in a socially and occupationally just workspace, they will be able to teach meaningfully and effectively. The findings highlight the role of teacher educators, schools, educational leaders, and decision-makers in creating the desired conditions for teachers. Teacher educators can design and implement programs and training which give a critical awareness on the role of teachers’ beliefs and self-efficacy on teachers’ performance. On the other hand, schools and school administrators should be willing to create a workspace in which teachers feel acknowledged and valued. They should also be able to create a balance between teachers’ workload and the expectations. Finally, educational decision makers should place polies that are facilitative of teachers’ work and provide professional development opportunities to share cutting the edge techniques and strategies in teacher-related research.

Despite its merits, the study has certain limitations that should be acknowledged. First, the number of participants in this study is not an exact representation of all EFL teachers in the Iranian context. The sample size of 120 participants may limit the generalizability of the findings to the entire population of language teachers. To enhance the external validity of future research, we recommend conducting studies with a larger and more diverse sample that includes a wider range of language teachers. Second, this study relied on self-report data, which is a commonly accepted and used data collection technique. However, it is important to recognize that self-report data may be subject to certain biases. For instance, participants may provide responses influenced by social desirability bias or have difficulty accurately recalling their experiences. To address this limitation and gain a more comprehensive understanding of language teachers’ experiences, we recommend incorporating additional data collection methods, such as interviews and reflective journals. These qualitative methods can offer rich insights into language teachers’ subjective experiences and perceptions, complementing the quantitative findings obtained through self-report questionnaires. Third, this study primarily relied on correlational analysis to report the results. While correlation analysis provides valuable insights into the relationships between variables, it does not establish causality or the direction of effects. To deepen our understanding of the causal relationships between occupational self-efficacy, satisfaction and meaning at work, and subjective well-being among language teachers, we acknowledge the need for further research employing longitudinal designs. Longitudinal studies can help investigate the temporal dynamics and directionality of these relationships, offering more robust evidence and stronger causal claims.

## Data availability statement

The original contributions presented in the study are included in the article/supplementary material, further inquiries can be directed to the corresponding author.

## Ethics statement

Ethical review and approval was not required for the study on human participants in accordance with the local legislation and institutional requirements. The patients/participants provided their written informed consent to participate in this study.

## Author contributions

SC wrote the first draft and contributed to subsequent revisions. FS collected the data and contributed to writing the first draft and revisions. IX designed the study, analyzed the data and supervised the project. All authors contributed to the article and approved the submitted version.

## Conflict of interest

The authors declare that the research was conducted in the absence of any commercial or financial relationships that could be construed as a potential conflict of interest.

## Publisher’s note

All claims expressed in this article are solely those of the authors and do not necessarily represent those of their affiliated organizations, or those of the publisher, the editors and the reviewers. Any product that may be evaluated in this article, or claim that may be made by its manufacturer, is not guaranteed or endorsed by the publisher.
